# Trend of Developing Aqueous Liquid and Gel Electrolytes for Sustainable, Safe, and High-Performance Li-Ion Batteries

**DOI:** 10.1007/s40820-023-01220-4

**Published:** 2023-11-06

**Authors:** Donghwan Ji, Jaeyun Kim

**Affiliations:** 1 School of Chemical Engineering, Sungkyunkwan University (SKKU), Suwon, 16419 Republic of Korea; 2 Department of Health Sciences and Technology, Samsung Advanced Institute for Health Sciences & Technology (SAIHST), Sungkyunkwan University (SKKU), Suwon, 16419 Republic of Korea; 3 Biomedical Institute for Convergence at SKKU (BICS), Sungkyunkwan University (SKKU), Suwon, 16419 Republic of Korea; 4 Institute of Quantum Biophysics (IQB), Sungkyunkwan University (SKKU), Suwon, 16419 Republic of Korea; 5 Present Address: Department of NanoEngineering, University of California San Diego, La Jolla, San Diego, CA 92093 USA

**Keywords:** Lithium-ion battery (LIB), Aqueous electrolyte, Gel electrolyte, Electrochemical stability window, Li dendrite

## Abstract

Current lithium-ion batteries (LIBs) rely on organic liquid electrolytes that pose significant risks due to their flammability and toxicity. The potential for environmental pollution and explosions resulting from battery damage or fracture is a critical concern. Water-based (aqueous) electrolytes have been receiving attention as an alternative to organic electrolytes. However, a narrow electrochemical-stability window, water decomposition, and the consequent low battery operating voltage and energy density hinder the practical use of aqueous electrolytes. Therefore, developing novel aqueous electrolytes for sustainable, safe, high-performance LIBs remains challenging. This Review first commences by summarizing the roles and requirements of electrolytes–separators and then delineates the progression of aqueous electrolytes for LIBs, encompassing aqueous liquid and gel electrolyte development trends along with detailed principles of the electrolytes. These aqueous electrolytes are progressed based on strategies using superconcentrated salts, concentrated diluents, polymer additives, polymer networks, and artificial passivation layers, which are used for suppressing water decomposition and widening the electrochemical stability window of water of the electrolytes. In addition, this Review discusses potential strategies for the implementation of aqueous Li-metal batteries with improved electrolyte–electrode interfaces. A comprehensive understanding of each strategy in the aqueous system will assist in the design of an aqueous electrolyte and the development of sustainable and safe high-performance batteries.

## Highlights

- This Review encompasses the role, requirement, and development direction of water-based electrolytes for sustainable, safe, high-performance Li-ion batteries.
- Water-based electrolytes (aqueous liquid and gel electrolytes) and their mechanisms are comprehensively summarized to widen the electrolyte electrochemical stability window and battery operating voltage and to achieve long-term operation stability.

## Graphical abstract

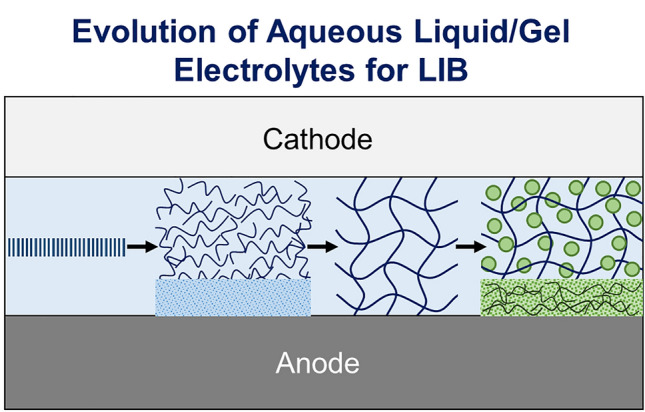

## Introduction

**Fig. 1:**
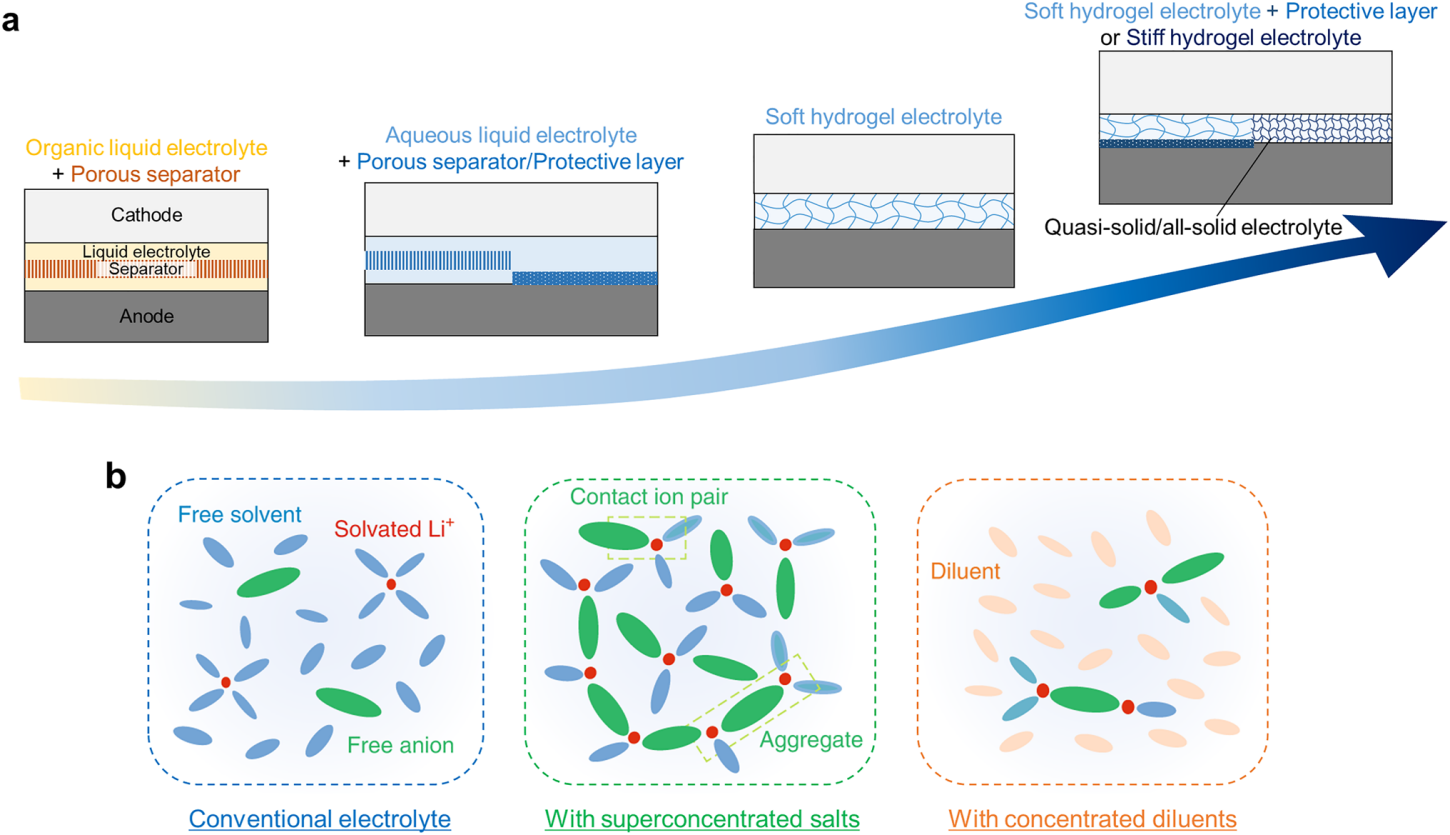
Trend of developing aqueous electrolytes. **a** Schematic of development direction from the combination of an organic liquid electrolyte and a porous separator toward a stiff hydrogel electrolyte playing the role of both electrolyte and separator. Quasi-solid/all-solid gel or polymer electrolytes are expected to replace the current combination of liquid electrolytes and separators.** b** Schematic of solvent (water molecule) and Li^+^ solvation structure of a conventional diluted electrolyte, an electrolyte with superconcentrated salts, and an electrolyte with concentrated diluents. Adapted with permission [28]. Copyright 2019, Springer Nature

Energy storage devices have become increasingly important in modern society. In particular, a rechargeable lithium-ion battery (LIB) is now an essential device and used in almost all electronics, such as portable devices and electric vehicles. This LIB generally consists of cathodes, anodes, separators, and electrolytes (Fig. 1a, the leftmost). A cathode is an electrode where reduction occurs (gain of electrons) while a battery discharges, whereas an anode is an electrode where oxidation occurs (loss of electrons). A separator is a type of liquid-permeable porous membrane keeping two electrodes apart to prevent physical contact and electric short circuits, and ionic charge carriers (Li-ions) move between the electrodes through pores. An electrolyte is a medium enabling Li-ion transport throughout the battery cell. Ions solvated by electrolytes move to the cathode or anode. Despite the considerable development in each battery component, the battery industry still suffers from several safety and environmental issues [1, 2]. Unexpected ignition and explosion of batteries in mobile phones and electric vehicles lead to severe accidents, and the generation of waste batteries and the toxicity of battery components are emerging as an environmental issue lately [3–5].

The ignition, explosion, and toxic pollution are usually provoked by the electrolyte of LIBs [2, 3]. The current LIB electrolyte is a liquid mixture comprising Li salts, additives, and organic solvents that are combined with cyclic carbonates for high Li-salt solubility and chain carbonates for low viscosity [6]. Because most of these organic solvents are highly toxic and flammable, pollution and explosion accidents easily occur when they are subjected to mechanical/thermal stresses or exposed to the open air. To address these issues, replacing the organic liquid electrolyte with water-based aqueous electrolytes could be a promising method [7–10]. However, current aqueous electrolytes also have limitations primarily due to the narrow electrochemical-stability window of water (1.23 V). Side reactions (e.g., hydrogen evolution reaction, HER) are unavoidable while ions are deposited on and stripped from the electrode within the existing aqueous electrolyte [11, 12]. These reactions do not enable the battery to have a wide electrochemical-stability window and high voltage and energy densities.

Since the introduction of the concept of 'water-in-salt' within an aqueous electrolyte for LIBs in 2015 [7, 8], substantial efforts have been dedicated to widening the electrochemical-stability window of aqueous electrolytes and achieving high-performance LIBs. The aqueous electrolytes were advanced by utilizing superconcentrated salts (e.g., LiTFSI) forming protective passivation layers, polymer additives assisting the role of superconcentrated salts, crosslinked polymer network acting as an additional protective layer, concentrated diluents (e.g., ionomer and polymer) replacing some salts until now. These advanced aqueous electrolytes, over the conventional aqueous electrolyte with high water content, demonstrated significant promise as substitutes for the current organic liquid electrolytes in LIBs (Fig. 1). As these studies continue to flourish and become more organized, we aim to present a compressive overview of the advancements in developing aqueous electrolytes for LIBs toward being sustainable and safe and having high performances (high-voltage and high energy densities). Accordingly, this Review will first commence by summarizing the roles and requirements of electrolytes–separators and then delineate the progression of aqueous electrolytes, encompassing both aqueous liquid and gel electrolyte development trends. Furthermore, we will discuss potential strategies for Li-metal-based aqueous LIBs utilizing quasi-solid or all-solid electrolytes (Fig. 1a, rightmost). The aqueous electrolytes are expected to be coextensive with solid ceramic or polymeric electrolytes in the future. Readers can refer to previous reviews describing detailed principles, mechanisms, and development histories of other battery components: electrode materials [13–16], organic liquid electrolytes [17, 18], separators [19–22], solid electrolytes [23–26], and battery-cell production [27].

## Basic Requirements for Developing Electrolytes

**Fig. 2:**
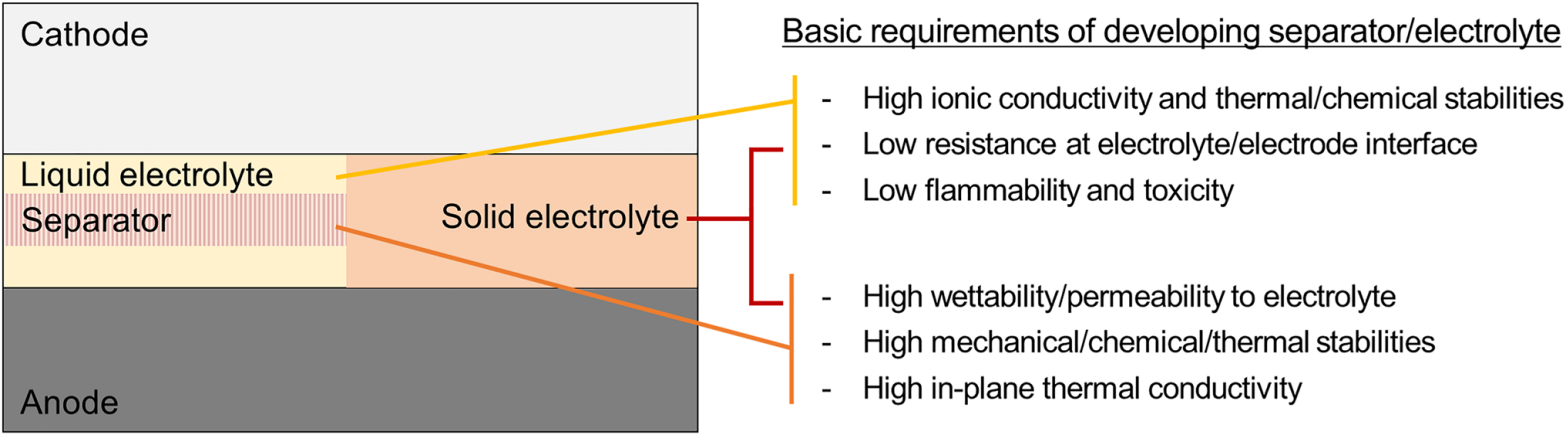
Basic requirements of developing a separator/liquid electrolyte or solid electrolyte

In the current battery structure, the electrolyte bridges two electrodes, and the separator membrane keeps two electrodes apart (Fig. 2). When one component cannot fulfill its role, the battery is typically immediately broken. At present, the combination of flammable organic liquid electrolytes and thin porous separators is vulnerable to mechanical and thermal stresses, which results in short circuiting, ignition, and explosion. In addition to direct external damage to the battery cell, internal overheating and subsequent gas generation in the cell also cause such accidents. The electrolyte and separator in most commercial batteries still suffer from several limitations, and it is challenging to determine a combination of electrolyte and separator with the balanced properties of high ionic conductivity; high thermal, chemical, and mechanical stabilities; high wettability and permeability; and low flammability, toxicity, and interfacial resistance with electrodes. Therefore, the role of these components and the conditions required for safe high-performance batteries should be clearly understood. In the case of the solid-state electrolytes (quasi-solid wet gels and all-solid dry ceramic or polymeric electrolytes), they should meet the requirements of both the separator and electrolyte.

In typical batteries, an electrolyte is developed based on several criteria for efficient battery performance and safe operation. The electrolyte is a medium for transporting ions and should have high ionic conductivity and high thermal and chemical stabilities. A few electrolyte constituents are intentionally induced to electrochemically decompose to form passivation layers on electrode surfaces (i.e., solid electrolyte interphase); however, most electrolyte constituents need to solvate ion salts stably without decomposition and gas generation during operation and at elevated temperatures. Moreover, battery performance can be high when the solvated ions quickly migrate to active materials in the electrodes. That is, easy ion transport and migration in electrolytes and at electrolyte–electrode interfaces with lower resistances are important. Furthermore, when the electrolyte is a stable and safe material without flammability, volatility, and toxicity, the batteries can be easily recycled and do not undergo severe accidents.

For separators preventing physical contact between two electrodes, only a separator with high mechanical, chemical, and thermal stabilities can serve its own purpose, and such features are the most important. Because the ions in the electrolyte pass through the separator, a separator with high wettability and permeability to the electrolyte enables efficient ion conduction between the electrodes. Moreover, when a separator has high in-plane thermal conductivity, internal heat can be effectively transferred to the outside, which suppresses local heat accumulation, battery-cell expansion, and even inhomogeneous ion precipitation and solid dendrite growth.

In addition to the aforementioned requirements, if metal anodes are employed, the electrolyte or electrolyte–separator combination should have the ability to suppress several anode issues, such as uneven electrodeposition, ion accumulation, and dendrite formation and growth, during repetitive charging and discharging [16, 29]; if not, a protective passivation layer should be coated on the anode instead. For aqueous electrolytes, the narrow electrochemical stability window of water and subsequent low energy density should be further addressed [2, 11].

Based on the advantages of aqueous electrolytes described in the Introduction, numerous studies have attempted to replace current organic liquid electrolytes with novel aqueous liquid or gel (hydrogel) electrolytes that can meet the aforementioned requirements. In the following sections, we will encompass an inclusive yet succinct account of LIBs employing an aqueous liquid or gel electrolyte with superconcentrated salts or concentrated diluents, and then introduce several strategies for developing electrolytes that implement stable and safe metal-anode batteries. Future electrolytes are expected to be water-based yet also suitable for a metal anode; therefore, we comprehensively summarize electrolyte studies that propose a method to control reactions on the electrode and improve the interface stability between electrolytes and electrodes.

## Aqueous Liquid and Gel Electrolytes with Superconcentrated Salts

Aqueous electrolytes offer competitiveness in cost, environmental friendliness (nontoxicity), thermal and chemical stabilities (nonvolatility and nonflammability), battery-power density, and fast charging rate (high ionic conductivity) compared to the organic electrolytes; however, the narrow electrochemical-stability window of water critically limits the battery-energy density. In particular, aqueous solvents and ionic salts generally cannot form solid electrolyte interphase (SEI) layers on the electrode surface; therefore, water-splitting reactions, hydrogen evolution reactions (HERs) on the anode, and oxygen evolution reactions (OERs) on the cathode readily occur, resulting in a narrow battery working voltage [30–32]. Moreover, the generated hydroxide and oxygen dissolved in the electrolyte corrode electrodes, and by-products and precipitations on the electrode hinder ion conduction between the electrolyte and electrode and power capability due to capacity fading.

**Fig. 3:**
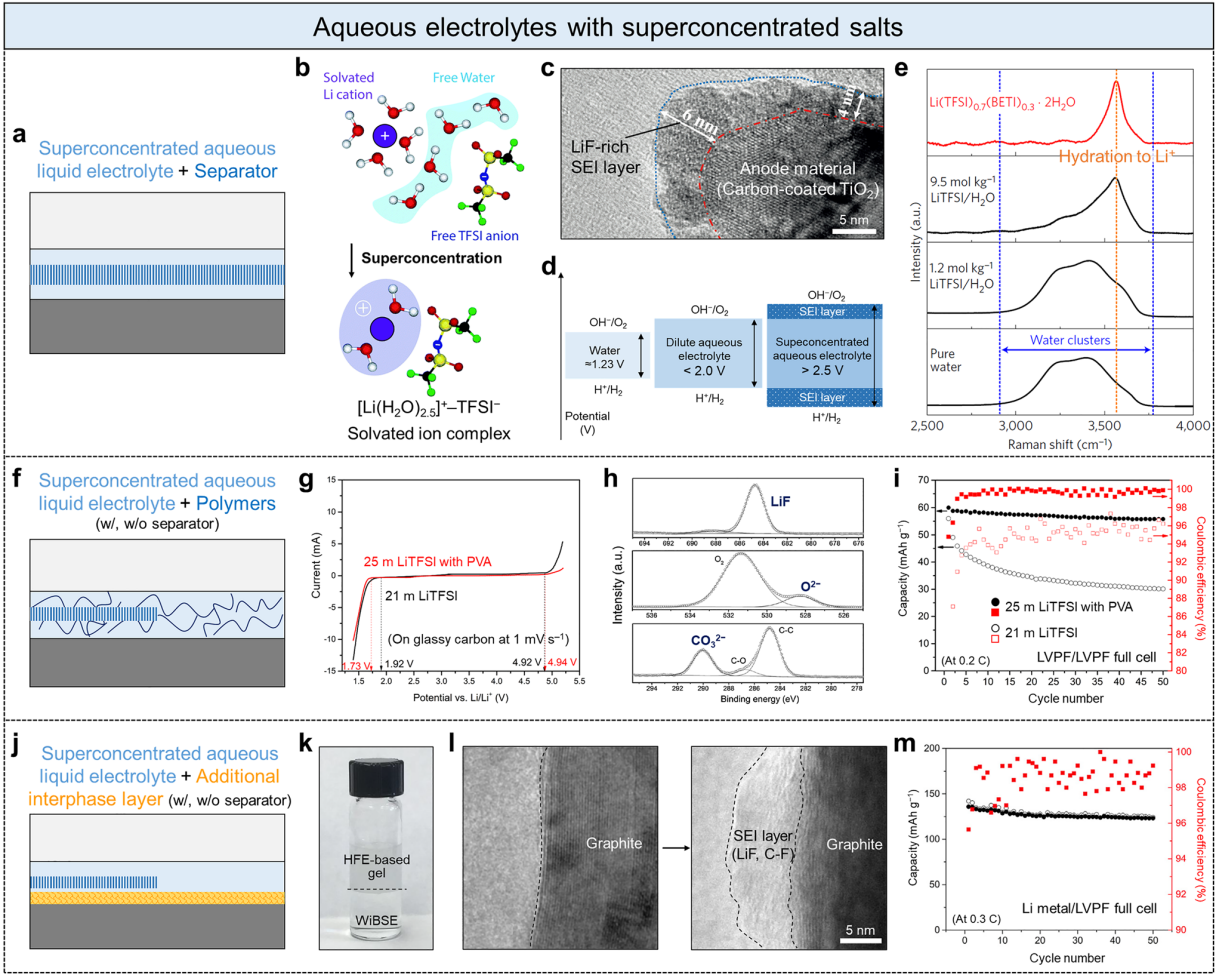
Aqueous electrolytes with superconcentrated salts. **a** Schematic of battery-cell component with superconcentrated aqueous liquid electrolyte and separator. **b** Solvation structure of typical diluted aqueous electrolyte and superconcentrated aqueous electrolyte. Adapted with permission [39]. Copyright 2018, The Royal Society of Chemistry. **c** Transmission electron microscope (TEM) image of the LiF-rich SEI layer formed along the carbon-coated TiO_2_ anode material. Adapted with permission [35]. Copyright 2016, Wiley–VCH. **d** Electrochemical stability window of each electrolyte. **e** Raman spectrum of superconcentrated aqueous electrolyte, diluted aqueous electrolytes, and pure water. Adapted with permission [36]. Copyright 2016, Springer Nature. **f** Superconcentrated aqueous liquid electrolyte with polymer additives. **g** Electrochemical stability windows of two different electrolytes, 21 m LiTFSI and 25 m LiTFSI containing PVA water stabilizer. **h** X-ray photoelectron spectroscope (XPS) F 1 s, O 1 s, and C 1 s spectra of LVPF anode at the fully delithiated state. **i** Cyclic stability and Coulombic efficiency of LVPF//LVPF full cell with two different electrolytes. Adapted with permission [40]. Copyright 2017, Wiley–VCH. **j** Superconcentrated aqueous liquid electrolyte and additionally coated interphase layer for a denser SEI formation. **k** Photograph showing the immiscibility between WiBSE- (21 m LiTFSI and 7 m LiOTf) and HFE-based gel. **l** TEM image showing the formation of dense SEI on graphite anode material. **m** Cyclic stability and Coulombic efficiency of Li metal//LVPF full cell consisting of Li metal anode coated with HFE-based gel. Adapted with permission [41]. Copyright 2017, Elsevier

To address these problems, electrolytes with superconcentrated salts, such as the water-in-salt electrolyte (WiSE) [7, 33, 34], water-in-bisalt electrolyte (WiBSE) [35], hydrate-melt electrolyte [36, 37], and monohydrate-melt electrolyte [38], were suggested in the last decade (Fig. 3). The core idea of these approaches is to reduce the number of free water molecules in the electrolyte and form a passivation SEI on the electrode. The slightly remaining water molecules are fastened and confined in ion hydration shells (Fig. 3b), and few salt anions decompose to form a LiF-rich SEI layer (Fig. 3c). As a result, the electrochemical stability window and battery operating voltage are widened (Fig. 3d).

In the first reported superconcentrated aqueous liquid electrolyte (i.e., WiSE) comprising 21 m lithium bis(trifluoromethane sulfonyl)imide (LiTFSI) (Fig. 3a), the number of water molecules coordinated in Li^+^ decreased to lower than 2.6 (i.e., water/LiTFSI ≤ 2.6), thereby resulting in the formation of [Li(H_2_O)_2.5_]^+^–TFSI^−^ solvated ion complex [7, 39] (Fig. 3b); all constituents, Li^+^, TFSI^−^, and H_2_O, are paired in each solvated complex [31, 42]. To increase the ion concentration over the saturation limitation of LiTFSI with a decrease in the number of water molecules, lithium trifluoromethanesulfonate (LiOTf), lithium bis(pentafluoroethanesulfonyl)imide (LiBETI), and lithium (trifluoromethanesulfonyl) (pentafluoroethanesulfonyl)imide (LiPTFSI) can be further added to form a WiBSE (21 m LiTFSI–7 m LiOTf) [35], hydrate-melt electrolyte (19.4 m LiTFSI–8.3 m LiBETI) [36, 37], and monohydrate melt electrolyte (22.2 m LiTFSI–33.3 m LiPTFSI) [38], respectively. Namely, water activity substantially declines because water molecules are isolated by bulky anions and fastened in Li hydration shells. In traditional dilute aqueous electrolytes, easily decomposable excess free water molecules exist and are identified as the broad peak of water O–H stretching resulting from diverse hydrogen bonds within water clusters through a Fourier-transform infrared (FTIR) or Raman spectroscopy analysis (Fig. 3e, black lines). In contrast, in superconcentrated electrolytes, free water molecules disappear, and only considerably strong specific interactions between the ions and water molecules in Li hydration shells exist. Such interactions are represented as a narrow sharp peak shifted toward higher energy in the Raman spectroscopy analysis (Fig. 3e, red lines).

More importantly, similar to an SEI formation in organic liquid electrolytes [43], superconcentrated fluorinated salt anions (TFSI^−^) can form LiF-rich SEI passivation layers at the anode (Fig. 3c). While the [Li(H_2_O)_2.5_]^+^–TFSI^−^ solvated ion complex is desolvated and the Li^+^ moves to the electrode, the released water molecules from the complex are reduced, generating hydrogen gas and highly reactive hydroxide (H_2_O + 2e^−^ → H_2_ + OH^−^) [39, 44]. The generated OH^−^ reacts with TFSI^−^, and TFSI^−^ is decomposed, which thereby forms F^−^ and fluorinated SEI (LiF-rich SEI) layers on the anode. The resultant SEI layer is an electron-insulating yet ion-conducting layer, unlike the ion-insulating by-product from corrosion in diluted aqueous electrolytes, which alleviates the hydrogen/oxygen evolution reactions. Then, Li^+^ reduction and Li deposition on the anode (Li^+^ + e^−^ → Li) from the solvated ion complex preferentially occur before further water reduction and TFSI^−^ degradation. Such SEI formation mechanism rarely works in the dilute aqueous electrolyte, in which anions (e.g., TFSI^−^) are not paired with Li^+^ and water as solvated ion complexes (Fig. 3b, top) [31, 42]. Consequently, the superconcentrated aqueous electrolyte with specific ion pairs as solvated ion complexes (Fig. 3b, bottom), which facilitate the reduction of anions to form LiF-rich SEI layers [28], can yield high-voltage batteries of over 2.5 V. Namely, the SEI layer pushes the hydrogen and oxygen evolution potentials beyond the thermodynamic stability limits of water (Fig. 3d), thereby resulting in the substantially larger operating voltage of the battery than that of batteries with pure water (≈1.23 V) or a diluted aqueous electrolyte (< 2.0 V).

To further exclude water molecules from the anode surface and promote the SEI formation to achieve long-term stability, polymers were additionally added and dissolved into the superconcentrated liquid electrolyte (Fig. 3f) [9, 40, 45, 46], or fluorinated additives immiscible with the co-existing electrolyte were coated on the anode surface (Fig. 3j) [9, 41]. In the former case (Fig. 3f), water-soluble hydrophilic polymers are generally added to the electrolyte as a water stabilizer. For example, polyvinyl alcohol (PVA) with abundant hydroxyl groups, which easily participates in Li solvation, increased the LiTFSI solubility from 21 to 25 m, decreased the number of water molecules, and contributed to the formation of a more robust SEI layer, which reduced the hydrogen evolution potential from 1.92 to 1.73 V (Fig. 3g) [40]. A strong LiF peak of the F 1 s XPS spectra confirmed the formation of LiF-rich SEI on a LiVPO_4_F (LVPF) anode surface (Fig. 3h). Moreover, strong O^2−^ and CO_3_^2−^ peaks of the O 1 s and C 1 s XPS spectra, respectively, revealed the formation of Li_2_CO_3_, which implies that the robust SEI layer is a mixture of LiF (51%) and Li_2_CO_3_ (49%). The resulting 25 m LiTFSI–PVA electrolyte improved the capacity cyclic stability and Coulombic efficiency of LVPF symmetric full cells (Fig. 3i). A rapid capacity reduction and relatively lower 96% Coulombic efficiency were observed in the 21 m LiTFSI electrolyte without the PVA additive, whereas the 25 m LiTFSI–PVA electrolyte slowed down the capacity decay and enabled the Coulombic efficiency to increase above 100%.

In the latter case (Fig. 3j), a hydrophobic highly-fluorinated-ether (HFE) additive was mixed with a small amount of polyethylene oxide (PEO) and LiTFSI to obtain a viscous gel paste (HFE-based gel, Fig. 3k), and this gel was applied and thinly coated on the anode surface [9, 41]. This hydrophobic HFE-based gel layer minimized the number of water molecules at the anode surface before the formation of the LiF-rich SEI layer from the superconcentrated salts during a few initial cycles. At the same time, HFE was consumed, thereby contributing to formation of a denser SEI layer comprising both inorganic LiF and organic C–F species (Fig. 3l). This dense SEI layer enabled the stable cycling of graphite or even Li-metal anode (Fig. 3m), which is a considerable difference compared to the conventional LiF-rich SEI layer (Fig. 3c) that was not robust enough to operate a battery comprising graphite or Li-metal anode.

**Fig. 4:**
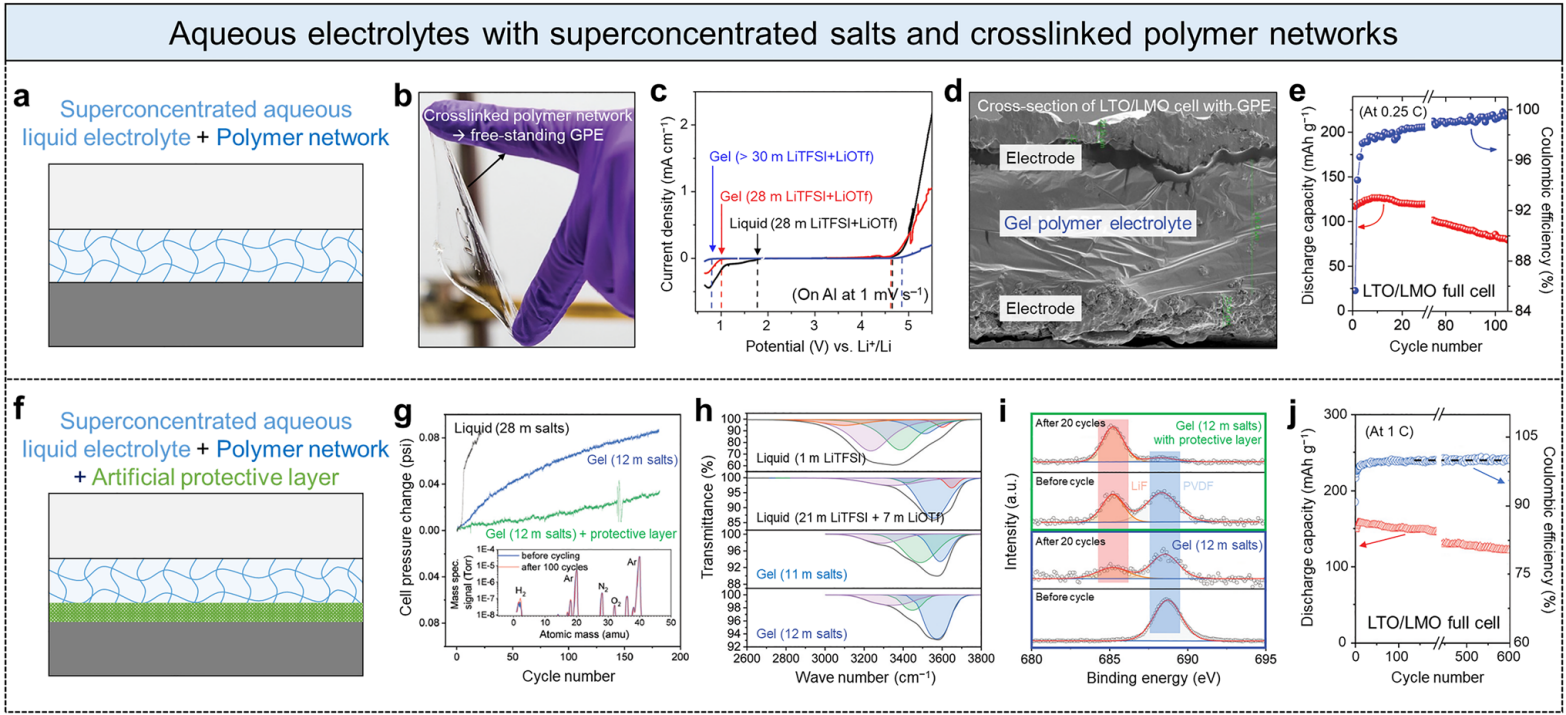
Aqueous electrolytes comprising superconcentrated salts and crosslinked polymer networks. **a** Schematic of battery-cell component with GPE. **b** Photograph showing a free-standing GPE. **c** Electrochemical stability windows of three different electrolytes, liquid WiBSE of 21 m LiTFSI and 7 m LiOTf, GPE containing 21 m LiTFSI and 7 m LiOTf, and GPE containing over 21 m LiTFSI and 7 m LiOTf. **d** Cross-sectional SEM image of LTO//LMO full cell with GPE. **e** Cyclic stability and Coulombic efficiency of LTO//LMO full cell with the GPE. Adapted with permission [47]. Copyright 2019, The Royal Society of Chemistry. **f** Superconcentrated aqueous GPE with artificial protective layer on anode surface. **g** Cell internal-pressure change by hydrogen-gas production in LTO//LMO full cell with three different electrolytes: liquid WiBSE of 21 m LiTFSI and 7 m LiOTf; GPE composed of 5.2 m LiTFSI, 5 m Pyr_13_TFSI, and 1.8 m LiOTf; and GPE with the protective layer. Inset: mass spectrometry graph. **h** FTIR spectra corresponding to the water O–H stretching bands of different electrolytes. **i** XPS F 1 s spectra of LTO anode surface in LTO//LMO full cell with and without the protective layer, before cycle and after 20 cycles. **j** Cyclic stability and Coulombic efficiency of LTO/LMO full cell with the GPE with additional protective layer. Adapted with permission [49]. Copyright 2020, The Royal Society of Chemistry

In addition to simple mixing polymer additives in the electrolyte (Fig. 3f), forming three-dimensional (3D) crosslinked polymer networks improved the electrolyte stability (Fig. 4a) [47, 48], and the combination of forming such crosslinked polymer networks and coating artificial protective layers was further employed (Fig. 4f) [49]. Because water molecules can be tightly bound to polymer networks [50–53], water activity and subsequent reduction can diminish more than water in the condition where no polymer network is present. Forming polymer networks with crosslinkers (Fig. 4a) is different from simple polymer additives, such as PVA and PEO (Fig. 3f). Although both are called gel polymer electrolytes (GPEs), the former is close to a free-standing viscoelastic hydrogel that can simultaneously act as a separator membrane, while the latter is a viscous yet still fluidic gel that cannot act as a separator membrane. Moreover, the SEI layer is an *in-situ* formed protective passivation layer generally comprising LiF and Li_2_CO_3_ during a few initial cycles (Fig. 3c), whereas the artificial protective layer is defined as an intentionally *ex-situ* coated passivation layer (Fig. 4f).

Langevin et al*.* fabricated a free-standing stretchable GPE (Fig. 4b), which comprised poly(ethylene glycol) methyl ether acrylate (MPEGA), 2-hydroxyethyl acrylate (HEA), and poly(ethylene glycol) diacrylate (PEGDA) with an 89:9:2 weight ratio and contained 21 m LiTFSI and 7 m lithium trifluoromethanesulfonate (LiOTf) (28 m LiTFSI + LiOTf) [47]. They also fabricated a GPE containing a significantly more concentrated LiTFSI by leveraging the high solubility of LiTFSI within the polymer gel (> 30 m LiTFSI + LiOTf). The GPE with significantly concentrated salts (blue line in Fig. 4c) exhibited better electrochemical stability than that of the GPE with lower salts (red line in Fig. 4c), and it was also superior to the conventional liquid electrolyte (black line in Fig. 4c) [47]. Moreover, this viscoelastic GPE could be neatly assembled with electrode materials without the interface detachment between the GPE and electrode materials (Fig. 4d), which helped maintain low interfacial resistance between each battery component. The final Li_4_Ti_5_O_12_//LiMn_2_O_4_ (LTO//LMO) full cell demonstrated stable operation over 100 cycles (at 0.25 C) (Fig. 4e).

Furthermore, an artificial protective layer can be precoated on an anode surface to further expel water molecules and promote the formation of a passivation interphase on the anode (Fig. 4f). For example, Zhang et al*.* first fabricated a viscoelastic stretchable GPE comprising bisphenol A ethoxylate dimethacrylate (BEMA) and poly(ethylene glycol)methyl ether methacrylate (PEGMA), and it contained a relatively lower amount of salts, 5.2 m LiTFSI, 5 m Pyr_13_TFSI, and 1.8 m LiOTf (total of 12 m salts) [49]. They also prepared a mixture by dissolving PEO, LiTFSI, and KOH with a weight ratio of 0.55:0.25:0.2, in methanol and cast it on an LTO anode to create an artificial protective (dried passivation) layer [49]. An internal gas pressure change of the LMO//LTO full cell during charge–discharge cycles demonstrated that GPE with 12 m salts substantially slowed down the hydrogen-gas production (blue line in Fig. 4g) compared with that of the typical superconcentrated aqueous liquid electrolyte (28 m salts, black line in Fig. 4g). This hydrogen gas production was further restrained by the protective layer (green line in Fig. 4g); the hydrogen gas was mainly generated during the SEI formation, and its amount was comparable with that generated in non-aqueous LMO//LTO full cells. The mass spectrometry verified only a small increase in hydrogen gas in the full cell during 100 cycles (Fig. 4g, inset). FTIR analysis also demonstrated the difference in electrolytes in amounts of free water molecules that are relevant to hydrogen-gas production (Fig. 4h). The diluted aqueous liquid electrolyte (1 m LiTFSI) had various O–H stretching peaks of weak energy that were similar to those of pure water, due to many hydrogen bonds within large water clusters. In contrast, the superconcentrated aqueous liquid electrolyte (21 m LiTFSI + 7 m LiOTf) showed shifts of O–H stretching peaks toward higher energy resulting from the limited number of water molecules and hydrogen bonds. For GPEs (with 11 or 12 m salts), the O–H stretching peaks became sharper in the high-energy region, representing that water activity and hydrogen-gas production are diminished. In addition, the protective layer promoted the SEI formation on the anode surface, which was revealed through XPS analysis (Fig. 4i). The protective layer comprising PEO, LiTFSI, and KOH immediately formed LiF on the LTO anode before an electrochemical cycle; PVDF was a binder material in the LTO. During 20 cycles, an F 1 s peak corresponding to LiF was intensified (red box in Fig. 4i), and an F 1 s peak corresponding to PVDF almost disappeared (blue box in Fig. 4i). Such immediate changes for 20 cycles, the increase in the LiF-peak and the decrease in the PVDF-peak, were not observed in the GEP condition without the protective layer. Consequently, the LTO//LMO full cell with the GPE and additional protective layer demonstrated an exceptional cyclic stability over 600 cycles (Fig. 4j). The capacity decay was only 0.034% per cycle and the average Coulombic efficiency was more than 99.9%. These values are comparable to those of conventional non-aqueous LIBs.

## Aqueous Liquid and Gel Electrolytes with Concentrated Diluents

Although the aforementioned superconcentrated electrolytes improve the battery operating voltage and long-term stability, the superconcentrated salts and the resulting solvation structure cannot completely avert water reduction. A study even demonstrated that hydrogen evolution originates primarily from water molecules in the solvation shell rather than from free water molecules [54]. Further, the formed LiF-rich SEI is not mechanically robust and thus repeatedly undergoes breaks and reforms. For that reason, water decomposition could not have been fully controlled in the aqueous electrolytes with superconcentrated salts. Moreover, only a limited number of lithium-salt types, which are generally expensive and toxic, satisfy the substantially high solubility required for superconcentration. The high cost and toxicity of the superconcentrated salts indeed blur the inherent advantage of aqueous electrolytes and consequently hamper their practical use and commercialization [55–58].

Concentrated diluents, which possess lower cost and toxicity concerns than concentrated salts, recently started being employed in an aqueous battery system to complement the superconcentrated salts [59–61]. In superconcentrated electrolytes, anion species considerably affect not only the formation of SEI layer but also the Li solvation structure and the number of water molecules coordinated in the solvation shell [39]. Similarly, a concentrated diluent, such as small molecules or polymers, in the electrolyte induces a different solvation structure by replacing water molecules with the diluent, and it decreases the total number of water molecules (Fig. 1b) [28]. The diluent facilitates the dissolution of Li salts (Li^+^ solvating) and also binds with water molecules, thereby weakening the Li^+^–water coordination strength (solvation strength) and the inter-water-molecule interactions (hydrogen bonds). The H–O bond strength in water molecules is strengthened accordingly, thereby alleviating the water reduction when the solvated ion complexes are desolvated for Li deposition [62]. Moreover, these aqueous electrolytes with a concentrated diluent generally exhibit advantages in terms of higher ionic conductivity as well as lower cost and toxicity than those with superconcentrated salts [59–61]. The ionic conductivity of aqueous electrolytes comprising ionomers, polymers, and small molecules as a diluent, which will be introduced right after, is in the range of a few milli siemens per centimeter (mS cm^−1^) that is the same magnitude as that of conventional non-aqueous electrolyte systems [22, 63, 64].

**Fig. 5:**
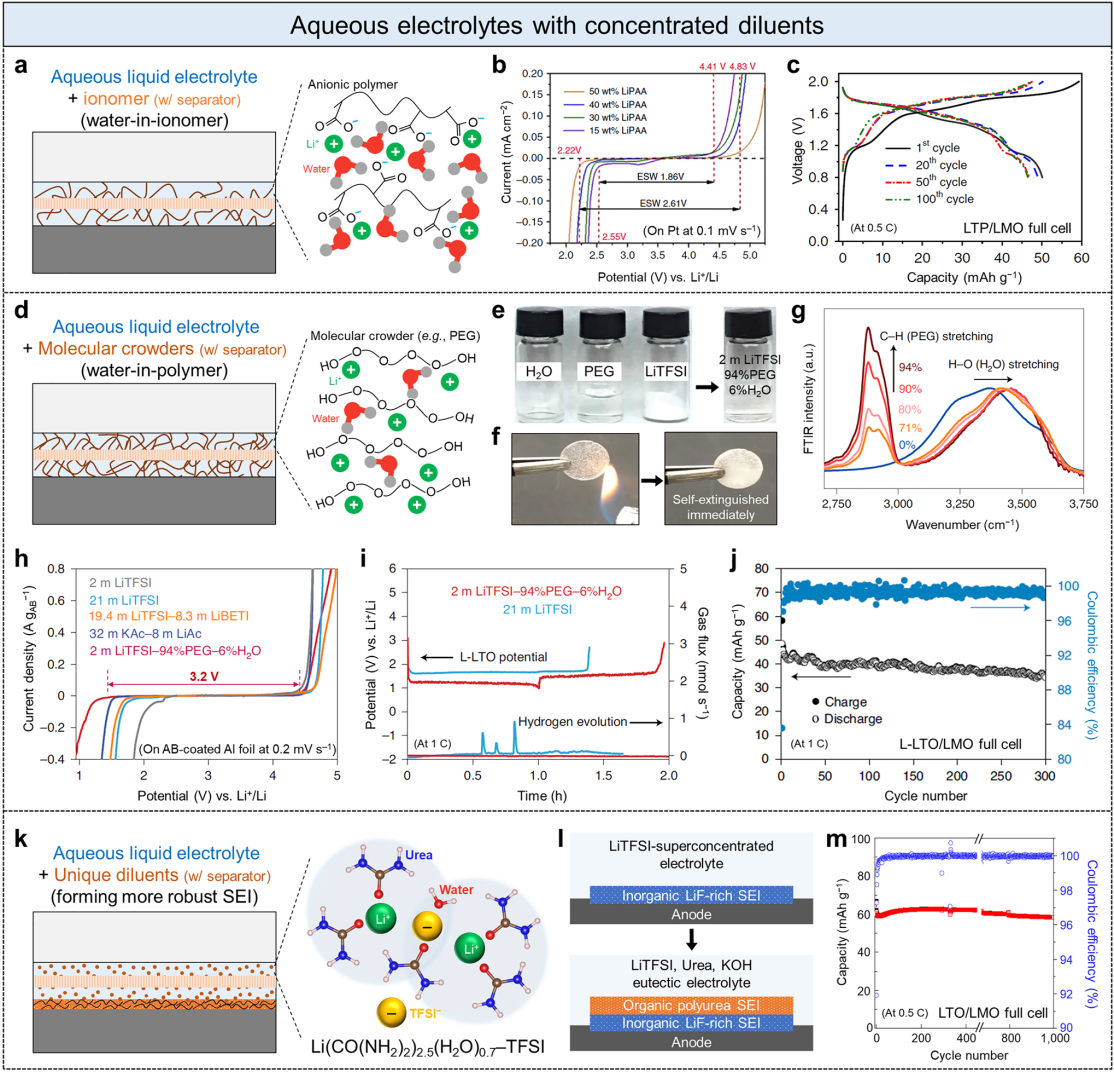
Aqueous electrolytes with concentrated diluents. **a** Aqueous liquid electrolyte with concentrated ionomers (water-in-ionomer) and its illustrative structure. **b** Electrochemical-stability windows of water-in-ionomer with different concentrations of ionomer. **c** Voltage profiles of LTP//LMO full cell with the water-in-ionomer electrolyte for selected cycles. Adapted graphs from [59]. **d** Aqueous electrolyte with concentrated molecular crowders (water-in-polymer or molecular crowding electrolyte) and its illustrative structure. **e,f** Photographs showing the fabrication and nonflammability of the molecular crowding electrolyte, respectively. **g** Normalized FTIR spectra of the aqueous liquid electrolyte with different concentrations of crowding polymer. **h** Electrochemical-stability windows and **i** online electrochemical mass spectroscopy (OEMS) of different aqueous liquid electrolytes. **j** Cyclic stability and Coulombic efficiency of L-LTO//LMO full cell with the molecular crowding electrolyte. Adapted with permission [60]. Copyright 2020, Springer Nature. **k** Aqueous liquid electrolyte with concentrated unique diluents that can form more robust SEI layers and its illustrative structure. **l** Schematic of formation of both inorganic LiF and organic polyurea SEI bilayers. **m** Cyclic stability and Coulombic efficiency of LTO//LMO full cell with the ternary eutectic electrolyte composed of LiTFSI, urea, and KOH. Adapted with permission [61]. Copyright 2022, Springer Nature

An ionomer, which comprises cationic Li^+^ and anionic polymer (e.g., polymer with acid groups), was introduced to prepare an inexpensive and nontoxic water-in-ionomer electrolyte (Fig. 5a) [59]. The water-in-ionomer electrolyte (LiPAA) comprised lithium hydroxide (LiOH), polyacrylic acid (PAA), and water instead of expensive and toxic fluorinated Li salts. Water molecules can interact with Li^+^ via its oxygen and with the carboxylate (COO^−^) of PAA via its hydrogens. In 50 wt% LiPAA electrolyte, which still contains 50 wt% water, the ratio of the number of water molecules to that of Li^+^ was only 4.33, and almost all water molecules were bound to Li^+^ or PAA with small binding energies (weak Li^+^–water coordination strength). Consequently, the water-in-ionomer electrolyte composed of even 50 wt% water exhibited a similar electrochemical stability window (2.6 V) to that of the superconcentrated electrolytes (Fig. 5b) and enabled a stable charge–discharge of LiTi_2_(PO_4_)_3_//LiMn_2_O_4_ (LTP//LMO) full cells over 100 cycles (Fig. 5c).

In addition, the concept of molecular crowders was employed in the electrolyte (Fig. 5d) [55, 60]; the molecular crowding electrolyte can be classified as a water-in-polymer electrolyte. Molecular crowding occurs routinely in living cells, where high concentrations of (macro)molecules reduce the solvent volume and particularly suppress water activity because water molecules are strongly confined to the molecular crowders. Some studies reported that polymer molecules can contribute more to the change in water activity, such as mobility and dipolarity/polarizability, compared with the contributions of inorganic salts [65–67]. In this scenario, the most important point to consider is strengthening the H–O covalent bond of each water molecule to increase the water molecule's electrochemical stability and suppress water reduction [68]. When a polymer is typically dissolved in liquid water, water molecules additionally make many hydrogen bonds with the polymer while still maintaining many hydrogen bonds between water molecules. However, when a high-concentration polymer is dissolved, and water molecules are relatively excluded in the mixture, despite the increasing number of hydrogen bonds between the polymer and water molecules, the total number of hydrogen bonds in the mixture can be considerably decreased, which reinforces the strength of the water molecules H–O bonding [69, 70].

Based on this, Xie et al. employed water-miscible, nontoxic, and cheap liquid polyethylene glycol (PEG), which has a strong Li^+^ solvating ability, and fabricated a non-flammable 2 m LiTFSI–94%PEG–6%H_2_O (weight percentage) aqueous liquid electrolyte with an ionic conductivity of 0.8 mS cm^−1^ (Fig. 5e, f) [60]. In the electrolyte containing high-concentration PEG, the bulk water volume was significantly reduced, thereby resulting in a decrease in the number of hydrogen bonds overall (particularly hydrogen bonds between water molecules). Moreover, with O^δ−^ of PEG (PEG–O^δ−^) as a hydrogen bond acceptor, which has higher electronegativity and acidity than them of O^δ−^ of water due to the electron-donating effect of the alkyl group in PEG, hydrogen ions of water (water–H^δ+^, hydrogen bond donor) were less transferred from water–H^δ+^ to PEG–O^δ−^. Thus, the remaining hydrogen bonds between PEG and water molecules were weaker than those between water molecules. Therefore, the H–O covalent bonds of water molecules were substantially strengthened by the molecular crowder PEG, which is necessary to suppress water decomposition. Such H–O bond strengthening was clearly verified as blue shifts (shifts toward higher energy) of the H–O stretching of water near a wavenumber of 3,400 cm^−1^ (Fig. 5g) and H–O bending of water near a wavenumber of 1,650 cm^−1^ in FTIR analyses. As a result, the 2 m LiTFSI–94%PEG–6%H_2_O aqueous liquid electrolyte (molecular crowding electrolyte) had an electrochemical stability window of 3.2 V superior to those of previous aqueous liquid electrolytes, and the lowest HER onset potential was notable (Fig. 5h, red line). The parasitic HER in a Li_1.3_Al_0.3_Ti_1.7_(PO_4_)_3_-coated Li_4_Ti_5_O_12_ anode and LiMn_2_O_4_ cathode (L-LTO//LMO) full cell was effectively suppressed by the molecular crowder (Fig. 5i, red line). This full cell exhibited cyclic stability over 300 cycles with an approximately 99% average Coulombic efficiency (Fig. 5j).

Despite the remarkable improvement in the electrochemical stability window through the above water-in-ionomer and water-in-polymer electrolytes, the hydrogen evolution on anodes may not be entirely suppressed for prolonged periods in actual cases due to the fundamentally unstable interface between the anode and electrolyte. Recently, a ternary eutectic aqueous liquid electrolyte comprising 4.5 m LiTFSI, 0.1 m KOH, CO(NH_2_)_2_ (urea), and water (urea:water = 8.6:1 volume ratio) was reported, and it had the ability to form a more robust SEI (originated by the polymerization of the diluent urea) [61]. Similar to the aforementioned molecular crowder, a large amount of urea was added as a diluent to obtain the liquid electrolyte (Fig. 5k). In the resulting electrolyte, urea decreased the number of water molecules in the Li^+^ solvation shell to 0.7, which was significantly lower than 2.6 in the WiSE (Fig. 3b). Water molecules were instead more coordinated with urea and the anion, and the weakening of the Li^+^–water coordination strength (solvation strength) and strengthening of the water H–O bond strength are assumed. Moreover, as opposed to the superconcentrated aqueous electrolytes in which OH^−^ generated by water reduction decomposed TFSI^−^ and formed the LiF-rich SEI, KOH as a catalyst in this new electrolyte facilitated TFSI^−^ decomposition to form LiF-rich SEI and urea polymerization on the anode (Fig. 5l). Namely, this ternary eutectic electrolyte formed both inorganic LiF and organic polyurea SEI bilayers that are more robust than the SEI of only inorganic compounds. As a result, the proposed electrolyte expanded the electrochemical stability window over 3.3 V and allowed LTO//LMO full cells to achieve a high average Coulobmic efficiency of 99.96% after 26 cycles and outstanding cyclic stability above 1,000 cycles (Fig. 5m). This performance was even maintained in a different form factor, such as a pouch-type cell, which demonstrated the potential for the practical engineering of electrolytes.

## Potential Strategies for Li-metal-based Aqueous LIBs

For Li-metal anode batteries, the electrochemistry for improving the battery operating voltage (suppressing water decomposition and widening electrochemical-stability window) and that for controlling reactions at anode surfaces and anode–electrolyte interfaces should be comprehensively considered. The charging–discharging chemistry (ion plating and stripping) on a metal-anode is different from the charging and discharging chemistry (ion insertion and desertion) in an anode material of particulate form. During the ion plating and stripping on the metal anode, stiff, hard, and sharp dendrites, which can pierce SEI layers, ex-situ coated protective layers, and even separator membranes, are readily grown along the metal surface as well as the water decomposition [29]. Thus, a homogeneous electrolyte flux and Li^+^ reduction are essential for flat Li plating without inhomogeneous Li nucleation and dendrite growth [71]. Since the water reduction and hydrogen evolution should be basically suppressed, modulating Li^+^ coordination structures, including Li^+^–water coordination (i.e., [Li(H_2_O)_n_]^+^ complex), and controlling [Li(H_2_O)_n_]^+^ complex transfers and Li deposition behaviors at the metal surface are the key to engineering aqueous Li-metal batteries. The intrinsic nature of Li metal, considerable instability (reactivity) to water, should be further considered. Because of these hurdles, studies on an aqueous electrolyte-based Li metal battery are actually lacking. Therefore, we here introduce and discuss potential strategies for implementing aqueous Li-metal batteries based on studies on aqueous Zn-metal batteries instead; the stable nature of Zn metal in water, unlike highly reactive and flammable Li metal in water, enables consistent and safe experiments and proofs-of-concept.

**Fig. 6:**
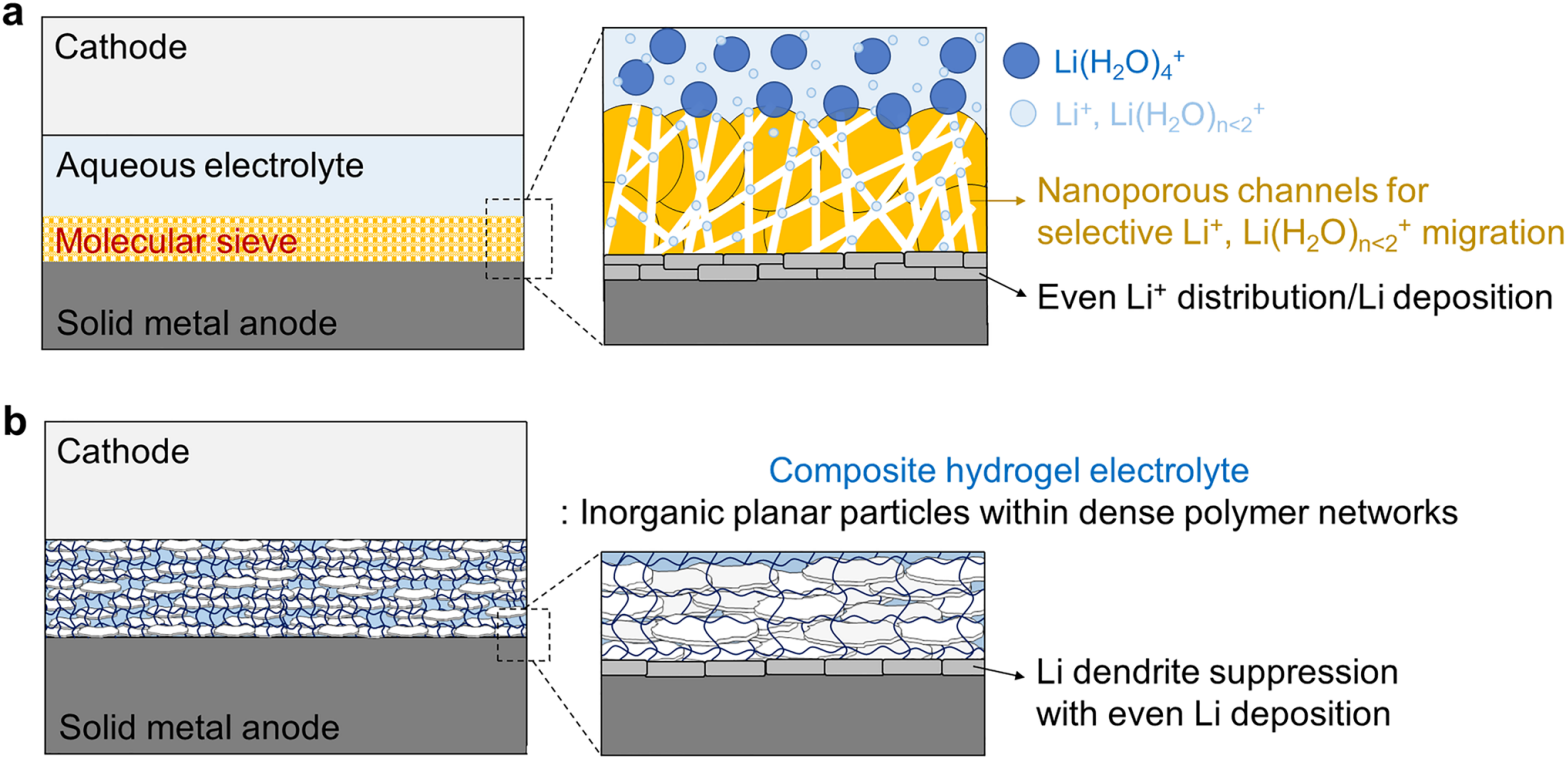
Potential strategies for aqueous Li-metal batteries. **a** Schematic of a molecular sieve serving as a nanoporous channel between the electrolyte and metal-anode interfaces. **b** Schematic of a mechanically outstanding composite hydrogel electrolyte comprising inorganic planar particles incorporated in dense polymeric networks

Expanding the concept of the aforementioned electrolytes with a decreased number of water molecules in the electrolytes and Li^+^ solvation shells utilizing concentrated salts/diluents, a molecular sieve located between the electrolyte and Li-metal would be able to offer better Li^+^ solvation structures to the Li metal (Fig. 6a). For examples in aqueous Zn-metal batteries, a nanoporous metal–organic framework ZIF-7 [72] or a mesoporous silica MCM-41 [73] was coated on the Zn metal as a molecular sieve. Similarly, sacrificial leaching of alloyed metal in Zn-metal was also introduced to create a nanoporous Zn structure (acting as a molecular sieve) at the metal–electrolyte interface [74]. The nanoporous channels of the molecular sieve restrict the migration path of ions and ion complexes owing to the confinement effect [75, 76]. The nanopores exclude large [Zn(H_2_O)_6_]^2+^–anion^2−^ solvated ion complexes, whereas substantially smaller ion complexes, such as [Zn(H_2_O)]^2+^–anion^2−^ and Zn^2+^–anion^2−^, can migrate to the Zn-metal under an electric field. This diminishes the degree of direct contact between the metal and water molecules desolvated from the solvation shell, which is a similar effect to that of concentrated salts/diluents. These molecular sieves and nanoporous channels in front of the metal surface can not only reduce water decomposition but also contribute to even ion distribution and deposition. In particular, a few nanometer-sized pores effectively strain the solvated ion complexes, and the sifted smaller ion complexes can have a weak ion–water coordination strength and be more easily and uniformly desolvated, thereby leading to homogeneous deposition on the metal [77, 78]. Therefore, the additional molecular sieving of the solvated ion complex generated by the above-described concentrated salts/diluents may be a strategy for implementing aqueous Li metal batteries.

In addition, the mechanical properties of the separator or gel electrolyte are critical factors in engineering the Li-metal battery owing to unexplained Li dendrite growths and short circuits. Stiff separators or solid electrolytes with a shear modulus approximately 1.8–2 times larger than that of Li metal can induce even and stable Li deposition on the metal surface, blocking the dendrite growth [29, 79]. Otherwise, moderate compressive stresses parallel to the Li-plating direction (cross-plane of laminated cell components), including stack pressure in battery cells, can rather direct dendrite growths toward the electrode, thereby accelerating short circuits [80]. The fact that specific mechanical force applied to Li dendrites in a solid electrolyte deflects the dendrite propagation was recently experimentally identified [80]. Compressive stresses, which are perpendicular to the Li-plating direction (opposed to the direction of Li-plating-induced pressure) and are larger than that of the Li-plating-induced pressure, can deflect the dendrite propagation direction, thereby averting short circuits; such compressive pressure applied from the electrolyte to the dendrite can be regarded as a shear force. Therefore, mechanically suppressing dendrite growth and propagation is practically important, and even if the dendrite was sharply grown, a separator or solid electrolyte should not be pierced by the dendrite.

Based on that, we assume that mechanically outstanding (especially high shear strength and modulus) quasi-solid hydrogel electrolytes (Fig. 6b) may suppress both water decomposition and dendrite growth. For example, organic–inorganic composite hydrogel electrolytes [81, 82] with highly densified hydrophilic polymer networks and concentrated salts or diluents are expected to modulate Li^+^–water coordination and suppress water reduction. Moreover, Li-dendrite growth, propagation, and direction can be managed by hard and stiff inorganic components incorporated in the densified polymer networks. The inorganic components can physically block the dendrite penetration and direct the dendrite toward relatively soft polymeric matrices [83]. If the dendrite begins to penetrate the dense polymer network, the polymer chains can push the dendrites. The induced force is a compressive stress perpendicular to the dendrite propagation direction, which leads to the deflection of dendrite growth. Accordingly, a quasi-solid composite hydrogel electrolyte, which exhibits both outstanding mechanical performances (solid-like high hardness and strength and high elastic/shear moduli within a unique structure) and good ionic and thermal conductivities with low internal resistance (no problem with separator wettability and permeability to electrolyte and local heat accumulation), can be a promising candidate for the metal-anode battery electrolyte [63].

## Conclusions and Outlook

Cutting-edge research on the aqueous LIBs demonstrated the potential of aqueous electrolytes for addressing the issues in ignition, explosion, and toxic pollution caused by organic electrolytes. According to these studies until now, the aqueous electrolytes were classified into three types (Fig. 1b): a conventional electrolyte containing a large number of free water molecules (solvent); an electrolyte with superconcentrated salts, in which all water molecules are solvated in Li shells forming Li(H_2_O)_n<2.5_; and an electrolyte with concentrated diluents, in which free water molecules are diminished by concentrated diluent molecules that are less expensive, toxic, and viscous than the concentrated salts, and the strengthening of H–O covalent bonds of water molecules or the accelerating of SEI formation are further induced by a unique diluent ability.

**Fig. 7:**
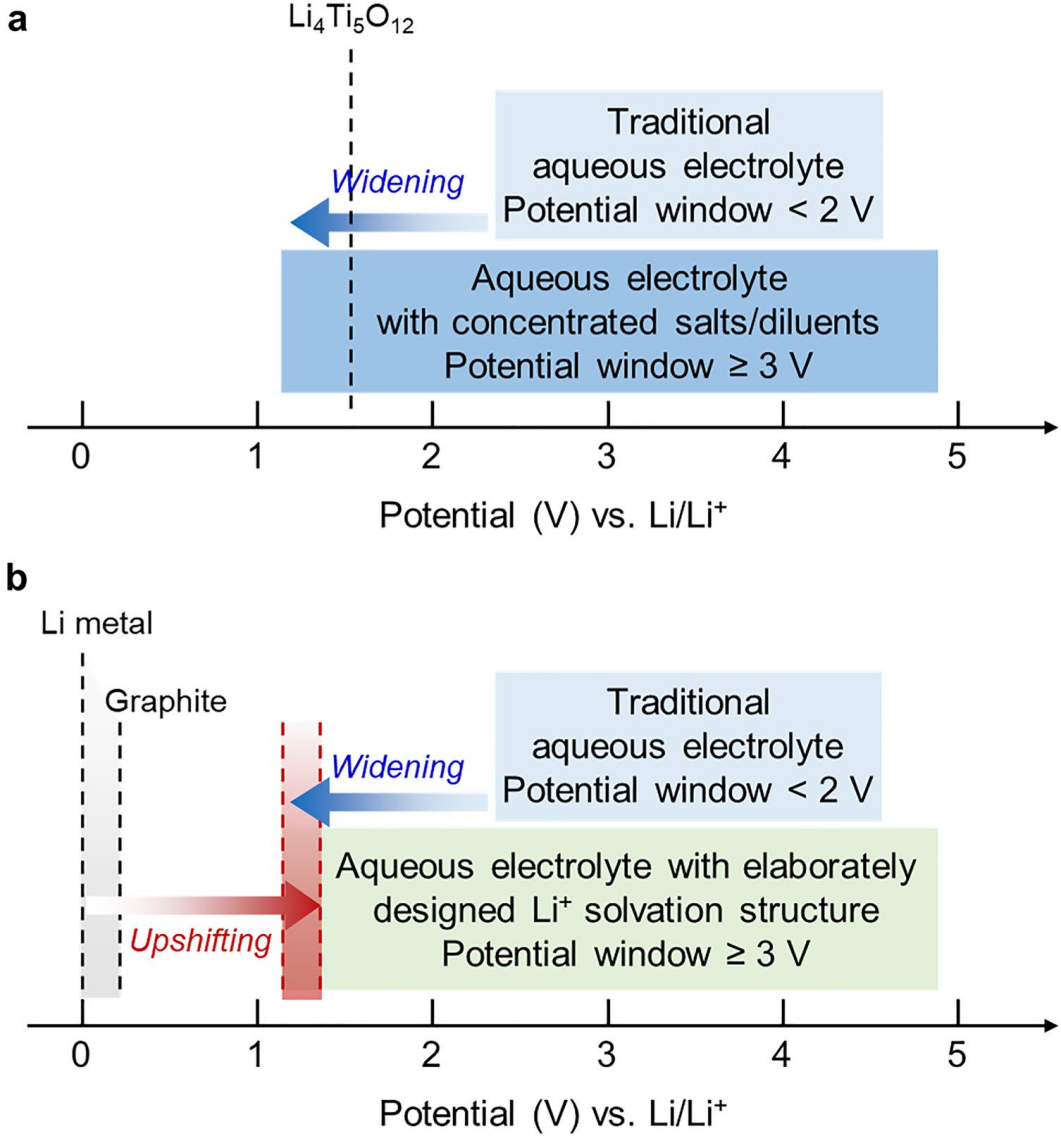
Electrochemical stability windows of electrolytes. **a** Aqueous electrolyte with concentrated salts/diluents, whose lower-end potential of the electrochemical-stability window is widened beyond the Li electrode potential, over the traditional aqueous electrolytes. **b** Aqueous electrolyte with intricately designed Li^+^ coordination structures for upshifting the electrode potential exceeding the lower-end potential of the electrolyte

We can summarize how to design the advanced aqueous electrolytes with a widened electrochemical-stability window whose lower-end potential is positioned at a more negative potential than the Li electrode potential (e.g., Li_4_Ti_5_O_12_) (Fig. 7a), as follows. First, the free water molecules (water clusters) must be removed, and all water molecules must participate in ion solvation. In this case, no hydrogen bonds exist between water molecules; therefore, the increased strength of the water H–O covalent bonds leads to less water decomposition. Second, the number of water molecules in the Li^+^ solvation shell must further decrease. Hydrogen evolution primarily originated when the water molecules were desolvated on the anode surface during the desolvation of Li(H_2_O)_n_ complex for the Li deposition. Additives can further reduce the number of water molecules by replacing H_2_O in the solvation shell, thereby weakening the bonding strength between ions and solvated water (Li^+^–water coordination strength, solvation strength) and strengthening the H–O covalent bonds of water molecules. Third, SEI formation must be promoted. For instance, superconcentarted fluorinated anions (e.g., TFSI^−^) in a solvated ion complex (e.g., [Li(H_2_O)_2.5_]^+^–TFSI^−^) form SEI layers along with the reduction of water molecules from the solvated ion complex at a few initial cycles (particularly, during the Li deposition at the anode). The SEI layer acts as a passivation layer, preventing water molecules from additional reduction and decreasing the onset potential for HERs. Additives can further facilitate/accelerate the formation of robust SEI layers that effectively prevent undesirable reactions such as water reduction.

On the other hand, the aforementioned advanced electrolytes comprising high-concentrated ingredients confront some challenges. These challenges include mediocre ionic conductivity, elevated viscosity, increased costs, ambiguous safety due to flammability concerns, and environmental impacts regarding toxicity and sustainability. Therefore, balancing these multifaceted properties based on understanding the above electrolyte chemistry would be essential for realizing practical aqueous LIBs.

In addition to widening the electrochemical stability window of the electrolyte, another approach, upshifting the electrode potential (e.g., Li metal or graphite) towards a more positive value than the lower-end potential of the electrolyte, is worth considering simultaneously (Fig. 7b) [84–86]. The upshifting of the electrode potential means that Li deposition can preferentially occur before electrolyte-component decomposition during charging, and the reactivity of the electrode (e.g., the reducing ability of Li metal to cause the reductive decomposition of the electrolyte) can be weakened. In other words, the electrode potential is positioned over the potential of water decomposition and hydrogen evolution. This approach can be realized by intricately manipulating Li^+^ coordination structures, such as solvation structures, Li^+^–water and Li^+^–anion interactions [84]. For example, electrolyte components with a poor ability to solvate Li^+^ (i.e., a weakly solvating electrolyte with low-polarity solvents) can facilitate high Li-electrode potential value, thereby positioning the electrode potential in the potential window of electrolyte [85]. In this perspective, balancing the nature of electrolytes that weakly solvate Li^+^ but moderately conduct Li^+^ would be necessary because weak solvation may lower the ionic conductivity of electrolytes. Optimizing the electrode potential to fabricate batteries with high-voltage and high-energy densities needs to be further considered at the same time.

A comprehensive understanding of how to control water reactivity (hydrogen/oxygen evolution), widen the electrochemical stability window of electrolytes, and manage Li deposition–dissolution reactions at the (metal) electrode are prerequisites for the rational design of aqueous electrolytes. In addition, we should consider a multitude of factors (such as ionic conductivity; wettability and permeability; thermal, chemical, and mechanical stabilities; flammability, volatility, and toxicity) for stepping toward commercialization of aqueous LIBs. Since magnificent research has well-established the principles and mechanisms for widening the electrochemical stability window, our focus can shift towards understanding the thermal, (electro)chemical, and mechanical stabilities of concentrated salts/diluents under various temperature/voltage/current conditions and their flammability, volatility, and toxicity from both the commercialization and sustainability perspectives.
